# Berberine and Etoposide Exert Synergistic Anticancer Effects in A431 Epidermoid Carcinoma Cells

**DOI:** 10.1021/acsomega.6c06421

**Published:** 2026-10-02

**Authors:** Zeynep Özlem Cinar, Valerija Šatrova, Akshay Kumar Vijaya, Milda Babonaitė-Aleksieju̅nė, Goda Glebavičiu̅tė, Aurelijus Burokas, Giulio Preta

**Affiliations:** † Institute of Biochemistry, Life Sciences Center, 54694 Vilnius University, LT-10257 Vilnius, Lithuania; ‡ Department of Biological Models, Institute of Biochemistry, Life Sciences Center, Vilnius University, LT-10257 Vilnius, Lithuania; § Institute of Biosciences, Life Sciences Center, Vilnius University, LT-10257 Vilnius, Lithuania

## Abstract

Berberine, a natural isoquinoline alkaloid derived from*Coptidis rhizoma* has long been used in traditional medicine and has recently attracted attention for its potential role in cancer therapy. Emerging evidence suggests that berberine can inhibit proprotein convertase subtilisin/kexin type 9 (PCSK9), a key regulator of lipid metabolism that has also been implicated in tumor biology. While the role of PCSK9 in cardiovascular diseases is well established, its contribution to cancer progression and therapeutic response remains incompletely understood. Here, we investigated whether berberine-mediated inhibition of PCSK9 could enhance the efficacy of chemotherapeutic agents. Berberine reduced PCSK9 expression at both mRNA and protein levels and increased LDLR abundance in A431 cells, which exhibit high basal PCSK9 expression. Functionally, berberine decreased cell viability in a dose-dependent manner and exhibited a strong synergistic interaction with etoposide, according to the HSA synergy score. Mechanistic analyses indicated that berberine enhances etoposide-induced cytotoxicity, at least in part by delaying the resolution of DNA-damage associated signals. Importantly, genetic silencing of PCSK9 recapitulated the sensitizing effect of berberine, while palmatine, a structurally related compound with lower efficacy in reducing PCSK9 levels, showed decreased synergy with etoposide, although evaluated under a different range of concentrations. Collectively, these findings support a role for PCSK9 in modulating cellular sensitivity to etoposide and suggest that berberine may act as a potential adjuvant in this context.

## Graphical abstract

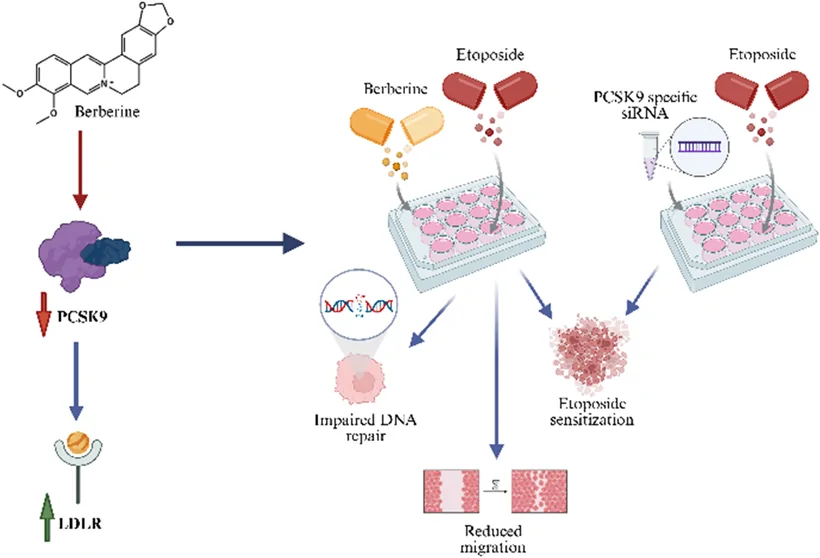

## Introduction

Lipids are key components of cell membranes, influencing cell growth, division, and intracellular signaling.^1^ Cholesterol, an essential component of these membranes, helps regulate membrane fluidity, facilitates cell adhesion to the extracellular matrix, and contributes to the initiation of several signaling pathways. The primary mechanism for removing cholesterol from the bloodstream is via low-density lipoprotein receptor (LDLR)-mediated uptake.^2^ Numerous studies have revealed a strong correlation between hyperlipidemia and cancer: to meet their high lipid requirements, cancer cells enhance lipid acquisition either by increasing dietary lipid uptake or by upregulating lipogenesis.^3–6^ Proprotein convertase subtilisin/kexin type 9 (PCSK9) is a key regulator in controlling cholesterol levels since its mature form, once secreted in the extracellular space, promotes LDLR degradation.^7–9^ Several therapeutic molecules that target PCSK9 have been developed over the years to treat hyperlipidemia as an effective alternative to statins for the prevention of cardiovascular diseases (CVD). PCSK9 monoclonal antibodies, evolocumab, and alirocumab bind to circulating PCSK9 molecules, thereby preventing their interaction with LDLR.^10,11^ siRNA Inclisiran represents another strategy to inhibit the internalization of LDLRs by specifically targeting the 3′ UTR of PCSK9 mRNA in the hepatocytes.^12,13^ Berberine, a natural plant alkaloid found in several herbs like *Berberis* species, has been shown to have lipid-lowering effects, partly through transcriptional inhibition of PCSK9 and consequent upregulation of LDLR expression.^14,15^ Berberine has also attracted significant interest in oncology due to its anticancer properties, both as a single agent or in combination with other compounds.^16–20^ Berberine functions as a natural chemosensitizer by modulating chemoresistance through various mechanisms, including activation of DNA damage, regulation of apoptosis, and downregulation of genes associated with multidrug resistance.^21–23^ Therefore, berberine is a valuable compound for understanding the connection between lipid metabolism and cancer due to its ability to modulate both processes simultaneously. It is reported to activate AMPK, a key regulator of cellular energy, which not only reduces lipid synthesis but also inhibits cancer-promoting pathways, including mTOR.^24,25^ Furthermore, by suppressing lipogenic enzymes such as SREBP-1c and ACC, berberine reduces lipid availability, disrupting cancer cell proliferation that relies on fatty acids for membrane formation and signaling. In this work, we investigated the potential use of berberine as an adjuvant in cancer therapy by targeting the lipid-regulating protein PCSK9. Specifically, we evaluated the cytotoxic effects of berberine on cancer cell lines with different levels of PCSK9 expression to determine whether its efficacy correlates with PCSK9 levels. A431, which expresses high levels of PCSK9, is particularly sensitive to berberine-induced cytotoxicity. By quantitative RT-PCR and ELISA analysis, we demonstrated a dose-dependent downregulation of PCSK9 levels and, as a consequence of this downregulation, an upregulation of LDLR protein levels. Combination treatment of berberine with different chemotherapeutic drugs in A431 cells revealed a synergistic effect with etoposide, as validated using the SynergyFinder software tool.^26,27^

A wound healing assay, an *in vitro* technique for assessing cell proliferation, showed that the combination of berberine and etoposide more effectively delayed the wound closure than either treatment alone, consistent with findings from previous studies.^28,29^ Analysis of the DNA damage response pathways showed that the berberine effect is mediated by the inhibition of DNA damage repair pathways, as previously observed in other cancer cell lines.^30,31^ To verify that PCSK9 suppression is essential for the observed synergistic effects, we employed a double approach using palmatine and specific PCSK9 siRNA. Palmatine, a compound structurally similar to berberine, is less effective at reducing PCSK9 expression.^32^ Combinatory treatment with etoposide resulted in a decrease in synergy scores (compared with berberine), indicating that PCSK9 inhibition is a key factor behind the synergistic effects. Similar results were observed when PCSK9 expression was downregulated by the use of siRNA, using non-targeting siRNA as negative control. Our study reveals that impaired DNA damage repair and effective PCSK9 inhibition are two key factors contributing to the synergistic effect of combined berberine and etoposide treatment, while also identifying PCSK9 as a novel possible regulator of cancer biology beyond its established role in cholesterol metabolism.

## Results

### PCSK9 Levels Differ among a Panel of Tumor Cell Lines

Emerging evidence suggests that PCSK9 may influence tumor progression through both lipid metabolism-dependent and independent mechanisms, including modulation of apoptosis, immune evasion, and proliferation pathways.^33–35^ Interestingly, PCSK9 expression can be influenced by several dietary and pharmacological agents, including berberine, a natural isoquinoline alkaloid. Berberine has been shown to downregulate PCSK9 expression and upregulate LDLR, thereby contributing to improved plasma lipid profiles and cardiovascular protection.^32,36^ The rationale of this study was to evaluate whether berberine, via inhibition of PCSK9 levels, can increase the cytotoxicity of common chemotherapeutic drugs (Figure 1A). For this reason, we selected a panel of four cell lines (A431, HepG2, PC-3, and HCT116) and analyzed PCSK9 protein levels using an ELISA kit. PCSK9 expression was highest in the A431 cell line, whereas the lowest expression level was observed in PC-3 cells. (Figure 1B).

**Figure 1:**
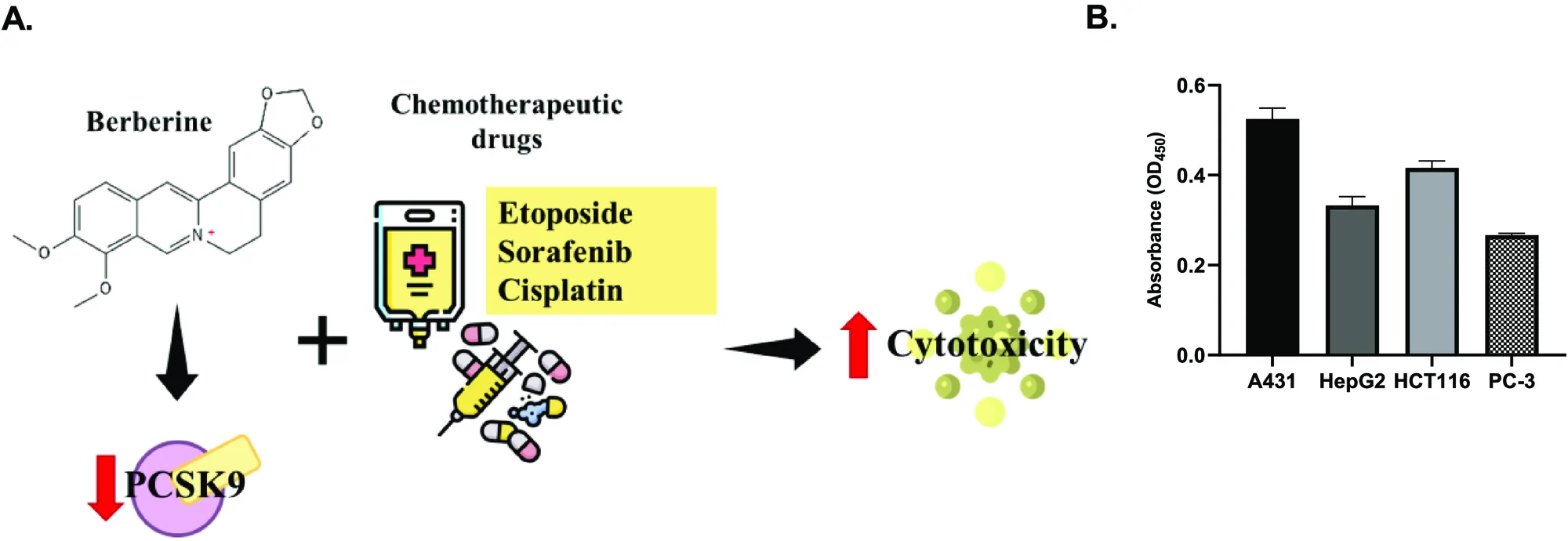
(A) Schematic illustration of the rationale of the study. (B) PCSK9 protein expression levels in A431, HepG2, HCT116, and PC-3 cell lines were determined by the PCSK9 ELISA Assay. Data are presented as mean ± SEM of three independent experiments.

### Berberine Exhibits Cytotoxic Effects on Different Cancer Cell Lines

To comprehensively assess berberine-induced cytotoxicity via distinct cellular mechanisms, two complementary assays were employed: the MTT assay, which evaluates mitochondrial metabolic activity as a measure of viable cells, and the LDH assay, which detects lactate dehydrogenase released into the culture medium as an indicator of membrane integrity loss due to cell damage or lysis. To minimize background LDH activity and reduce interference from serum-derived enzymes, the 10% FBS culture medium was replaced with 1% FBS medium before berberine treatment. This setting was maintained across all other berberine experiments. Cells were exposed to a broad concentration range of berberine (10, 25, 50, 100, and 200 μM) for 24 h to evaluate dose-dependent cytotoxic responses, as previously conducted in studies investigating the cytotoxic effects of berberine.^37,38^ Berberine demonstrated a consistent behavior by reducing cell viability in a dose-dependent manner in both assays (Figure 2). Among the tested cell lines, only in A431 cells, berberine decreased the viability under 50% upon treatment with 100 μM and 200 μM doses (Figure 2C). Furthermore, LDH assay results revealed a substantial increase in cytotoxicity in A431 cells, with approximately a 5-fold elevation in LDH release compared to the control group at 200 μM berberine (Figure 2C). Given their pronounced sensitivity to berberine and elevated PCSK9 expression, A431 cells were selected for further mechanistic evaluations.

**Figure 2:**
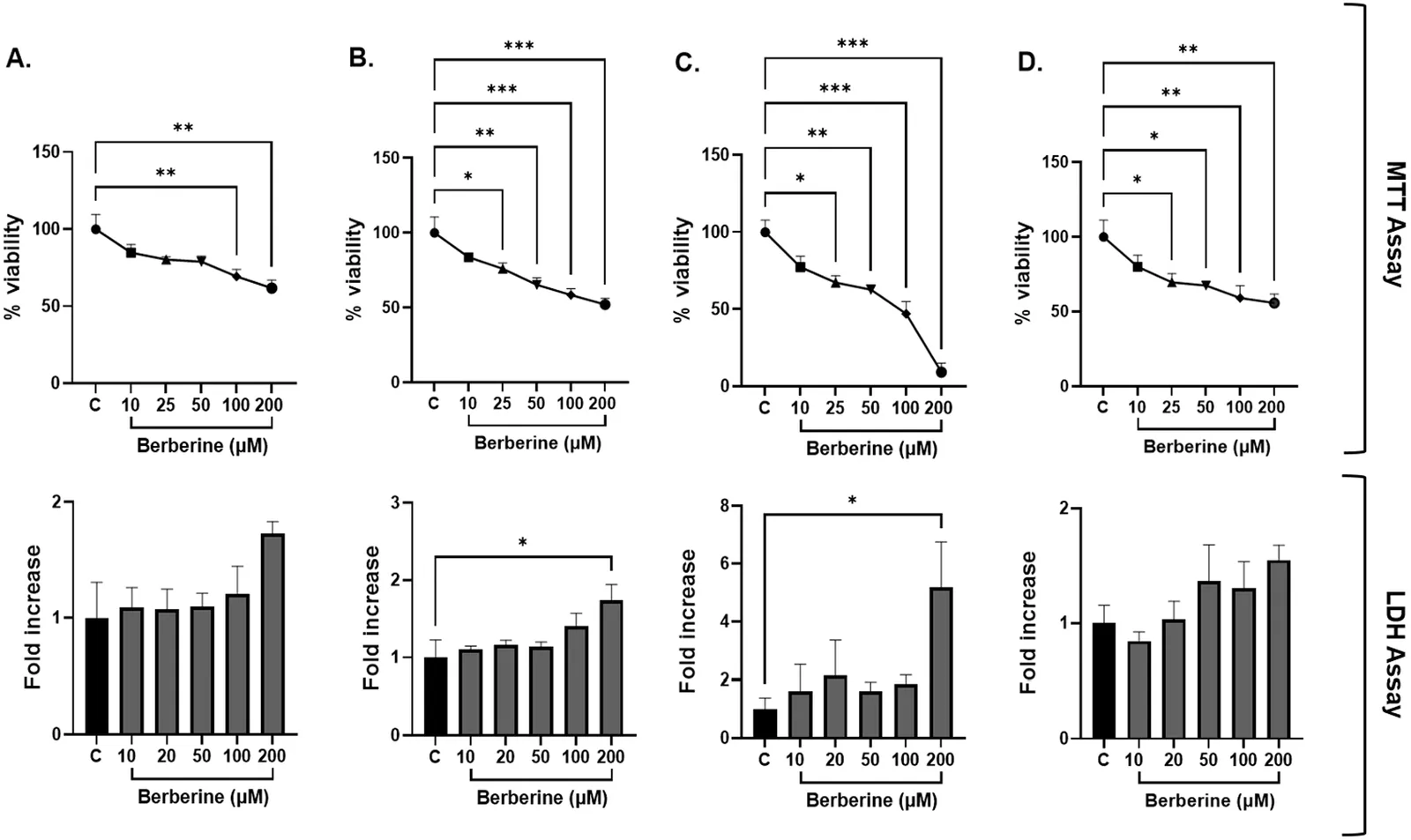
Effects of the berberine on the cell viability of (A) HepG2, (B) PC-3, (C) A431, and (D) HCT116 cell lines determined by MTT and LDH assays after 10, 25, 50, 100, 200 μM treatments for 24 h. C: Control. Data are presented as mean ± SEM of three independent experiments, and statistical analysis was performed using one-way ANOVA and Dunnett’s post hoc test. *p ≤ 0.05, **p ≤ 0.01, ***p ≤ 0.001.

### Berberine Treatment Decreases the PCSK9 and Increases the LDLR Levels in A431 Cell Line

To further elucidate the relationship between PCSK9 expression and the cellular response to berberine in A431 cells, both the transcriptional levels of PCSK9 and LDLR, as well as their post-transcriptional levels, were assessed. Quantitative RT-PCR analysis demonstrated that berberine treatment significantly downregulated PCSK9 expression at mRNA and protein levels in a dose-dependent manner (Figure 3A,3B). In parallel, immunofluorescence staining revealed a significant increase in LDLR protein expression after 24 h of berberine exposure, supporting the notion that PCSK9 negatively regulates LDLR availability (Figure 3C). In contrast, LDLR mRNA expression was notably reduced following berberine treatment in a dose-dependent manner (Supporting Information, Figure 1A). This discrepancy between mRNA and protein levels suggests a post-transcriptional stabilization mechanism of LDLR, likely resulting from decreased PCSK9 activity. Similar observations have been reported in statin-treated models with PCSK9 downregulation, where enhanced LDLR protein accumulation occurred independently of transcriptional activation.^39^

**Figure 3:**
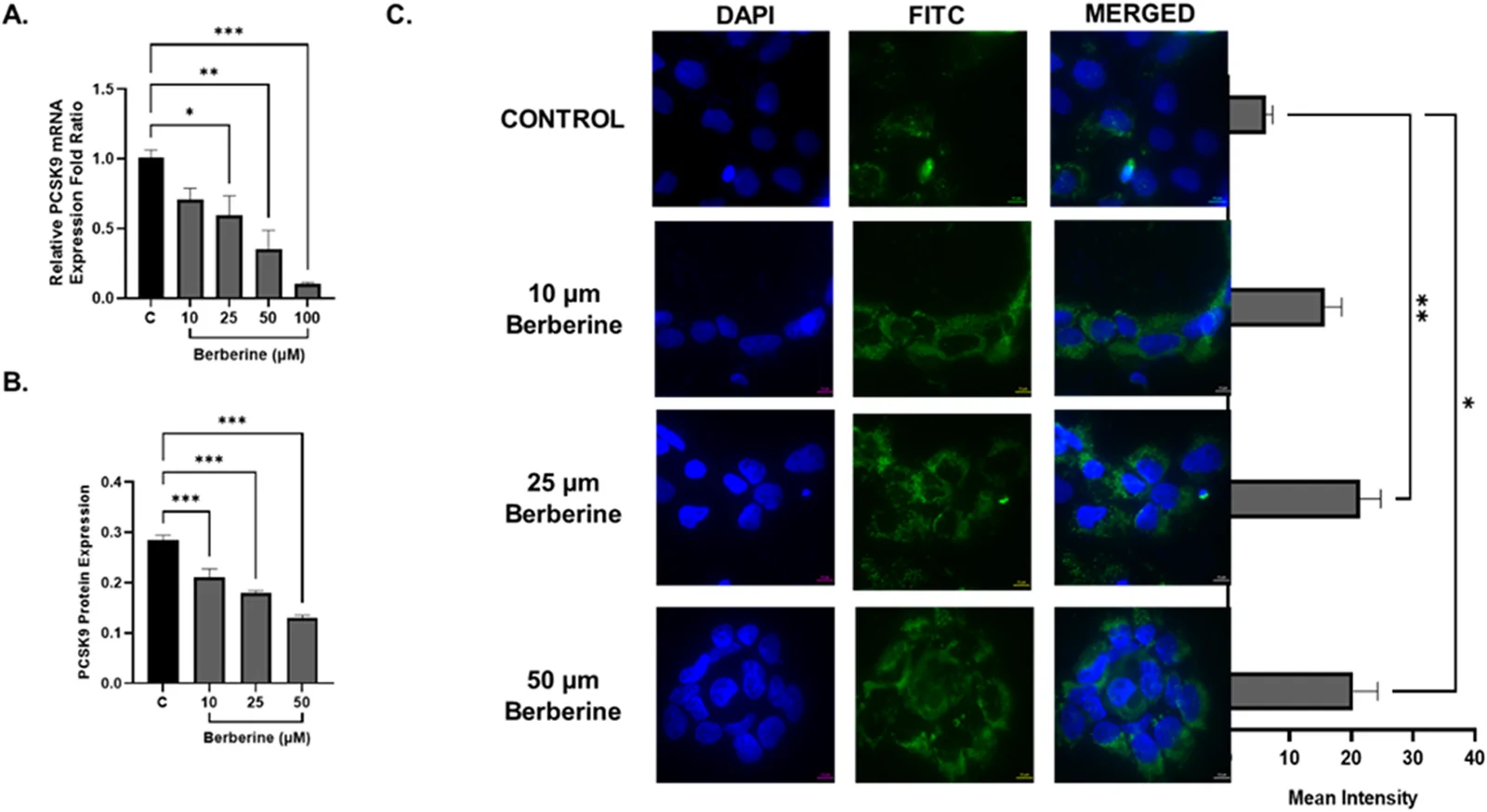
Effects of berberine on PCSK9 expression at the mRNA and protein levels and on LDLR expression at the protein level in A431 line. Expression levels of PCSK9 on (A) mRNA and (B) protein levels in A431 cells following berberine treatment ((A) 10, 25, 50, 100 μM, 24 h; (B) 10, 25, 50 μM, 24 h), normalized to untreated controls. (C) Quantification of LDLR expressions and representative immunofluorescence images showing LDLR (green-FITC) expression in A431 cells following berberine treatment (10, 25, 50 μM, 24 h). Nuclei were counterstained with DAPI in blue. C: Control. Scale bars: 10 μm. Data shown are mean ± SEM of three (A) or four (B, C) independent experiments, and statistical analysis was performed employing one-way ANOVA and Dunnett’s post hoc test. *p ≤ 0.05, **p ≤ 0.01, ***p ≤ 0.001.

### Combination Treatment with Berberine Enhances the Cytotoxicity of the Chemotherapeutics Etoposide and Sorafenib in the A431 Cell Line

Following the identification of A431 cells as the most responsive cell line to berberine treatment, further cytotoxicity assessments were conducted to investigate the potential synergistic effects of berberine in combination with standard chemotherapeutics, including etoposide, sorafenib, and cisplatin. MTT assays were performed using berberine alone (5 or 10 μM), each therapeutic agent alone (5 or 10 μM for etoposide and cisplatin; 1 or 2.5 μM for sorafenib), and all possible combinations at the indicated concentrations. Concentrations for each chemotherapeutic drug were chosen based on a dose–response curve, ensuring that cell viability remained above 50% (Supporting Data, Figure 2). Furthermore, for berberine, the doses used in combination treatments were selected based on DAPI staining results, which reflect the extent of nuclear damage. In this assay, 10 μM berberine did not induce significant DNA fragmentation compared to the control group, whereas 25 and 50 μM treatments resulted in pronounced nuclear condensation and fragmentation (Supporting Data, Figure 1B). The potential synergistic interactions between berberine and chemotherapeutic agents were evaluated using SynergyFinder 2.0 software. Two reference models were applied: the Bliss independence model and the Highest Single Agent (HSA) model. The Bliss model assumes that drugs act independently and estimates the expected combined effect based on the probability of independent action. In contrast, the HSA model compares the observed combination effect to the impact of the most active single agent at the corresponding concentrations.^27^

The combination of berberine and etoposide produced the strongest cytotoxic effect among all groups tested. At the highest concentration for both (10 μM berberine and 10 μM etoposide), A431 cell viability was reduced to 55%, significantly surpassing the effects of each drug alone (72.5% for berberine and 69% for etoposide). This enhanced cytotoxicity was reflected by the synergy score, with the Bliss and HSA models showing values of 0.776 and 12.913, respectively, indicating a synergistic interaction, particularly in the red area (Figure 4A). Additionally, acridine orange/ethidium bromide dual staining confirmed that treatment with the highest concentrations of both agents (10 μM berberine and 10 μM etoposide) resulted in approximately 40.3% A431 cell death, which was significantly greater than that observed with either treatment alone (25% for berberine and 17% for etoposide) (Supporting Data, Figure 3). The combination of berberine and sorafenib also exhibited an enhanced cytotoxic effect in A431 cells, with Bliss and HSA scores of 0.066 and 10.723, respectively, while the berberine and cisplatin combination revealed the least effective outcome, with the calculated Bliss and HSA synergy scores of −2.504 and 2.353, respectively (Figure 4B,4C). Notably, the combination of etoposide or sorafenib with berberine showed a more pronounced synergistic interaction according to the HSA model than to the Bliss model, suggesting that these combinations substantially outperform the more active single agent. Based on these findings, we selected the higher concentrations of berberine and etoposide (10 μM each) for further investigation into the underlying mechanisms of berberine’s effects in A431 cells.

**Figure 4:**
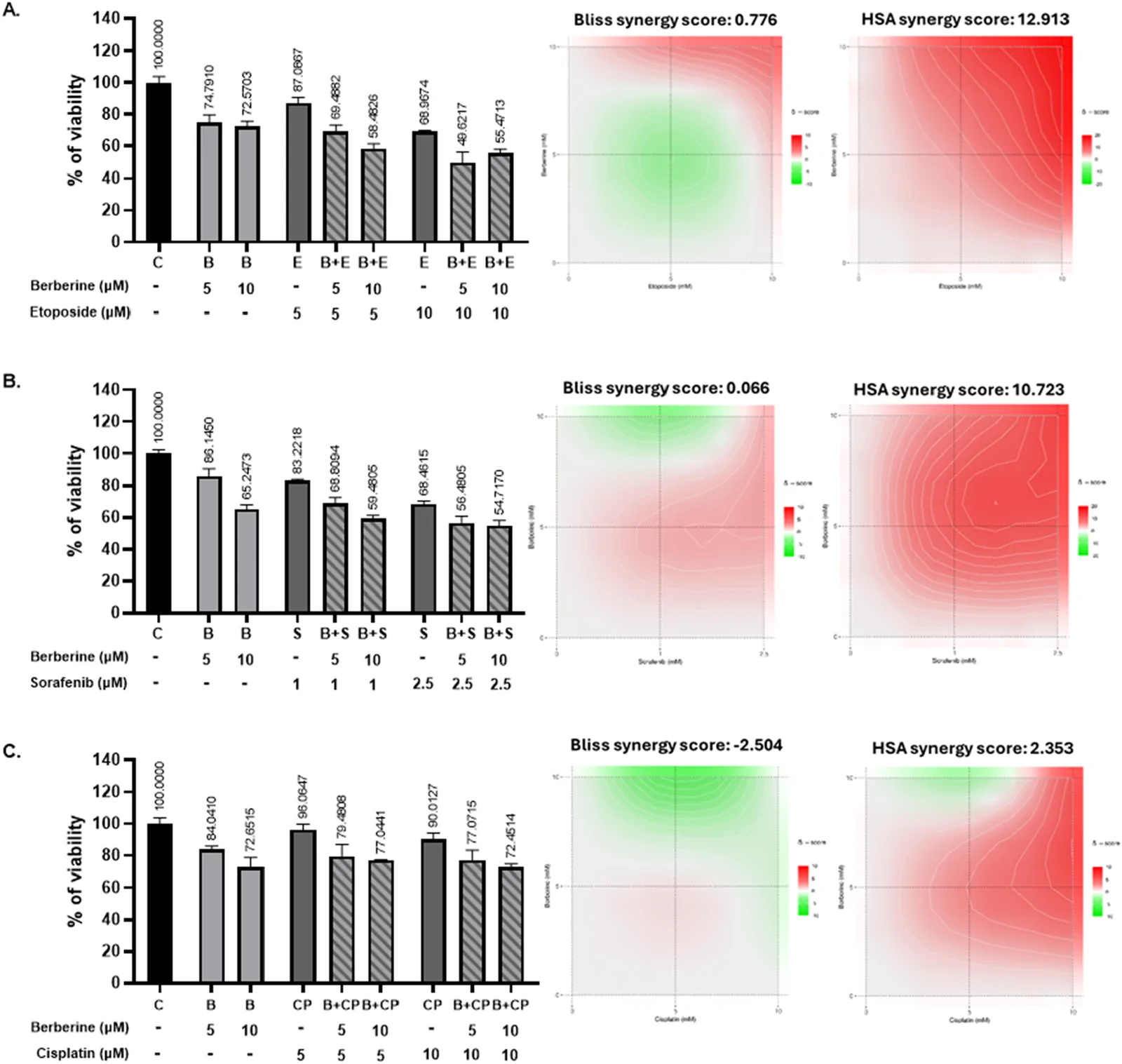
Effects of the combination treatment of therapeutics with berberine on the viability of A431 cells at 24 h and the assessment of potential synergistic effects, using the Bliss (left) and HSA synergy scores (right). (A) Treatment of berberine alone (5, 10 μM), etoposide alone (5, 10 μM), and the combination treatment of berberine and etoposide. (B) Treatment of berberine alone (5, 10 μM), sorafenib alone (1, 2.5 μM), and the combination treatment of berberine and sorafenib. (C) Treatment of berberine alone (5, 10 μM), cisplatin alone (5, 10 μM), and the combination treatment of berberine and cisplatin. All possible combinations of the indicated concentrations were evaluated. C: Control, B: Berberine, E: Etoposide, S: Sorafenib, CP: Cisplatin. Data shown are mean ± SEM of three (Etoposide, Cisplatin) or four (Sorafenib) independent experiments.

### Combination Treatment with Berberine Enhances the Antiproliferative Effects of Etoposide and Delays the Wound Closure

To assess the potential antiproliferative effects of the combination of berberine and etoposide in A431 cells, a wound healing assay was conducted at different time points. Quantification of wound closure after 24 h revealed that the combination treatment significantly reduced the wound closure by approximately 1.8-fold compared to either agent alone, both of which displayed similar levels of inhibition (Figure 5). These findings suggest that berberine and etoposide may exert a cooperative antiproliferative effect when used in combination. To our knowledge, only a limited number of studies have examined berberine alone or in combination with chemotherapeutic agents using wound healing assays. For instance, MG-63 osteosarcoma cells treated with berberine and cisplatin exhibited significantly reduced migration compared to single treatments.^19^ In another study, migration assays such as wound healing and transwell assays were used to show that berberine alone inhibits wound closure in colon cancer cell lines, including HT-29, SW-480, and HCT-116.^29^ Our study builds on these observations by evaluating the impact of berberine alone and in combination with etoposide on delayed wound closure of A431 epidermoid carcinoma cells. However, since cell viability was also reduced under these conditions, the observed effect may reflect both decreased cell migration and reduced cell proliferation.

**Figure 5:**
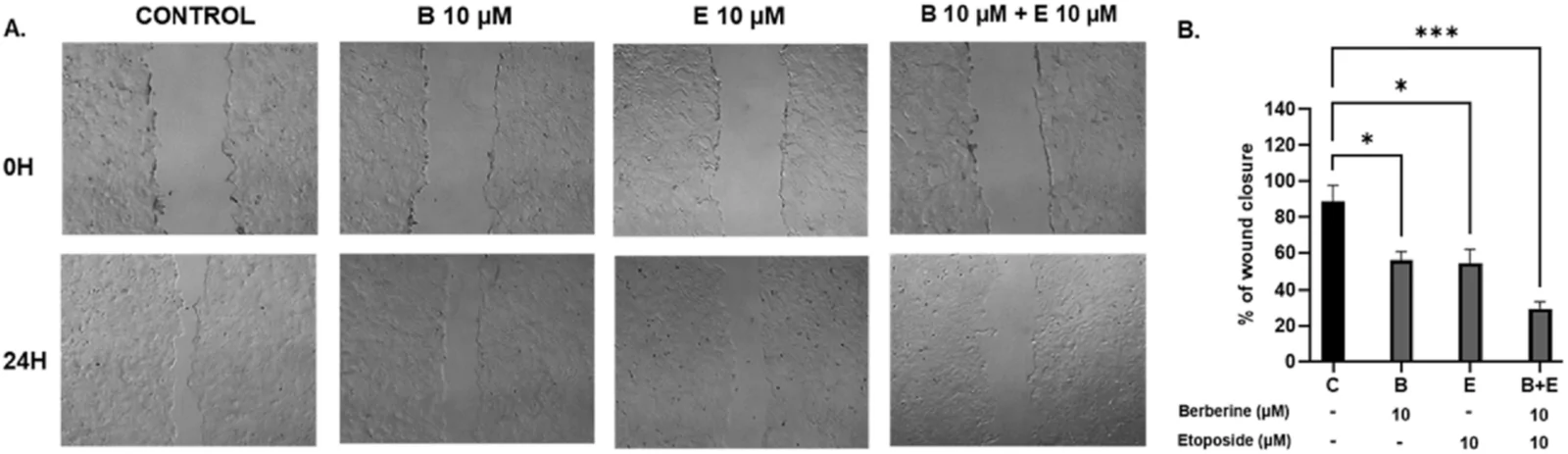
Wound healing assay showing the effect of berberine alone (10 μM), etoposide alone (10 μM), and the combination of berberine and etoposide (B 10 μM + E 10 μM) on the migration ability of A431 cells. (A) Representative images of wound closure at 0 and 24 h postscratch in control and treated groups. (B) Quantification of wound closure is expressed as a percentage of the initial wound area. C: Control, B: Berberine, E: Etoposide. Data shown are mean ± SEM of three independent experiments, and statistical analysis was performed employing one-way ANOVA and Dunnett’s post hoc test. *p ≤ 0.05, ***p ≤ 0.001.

### Elevated γH2AX Intensity Suggests Impaired Resolution of DNA Damage upon Combined Treatment

To further explore the mechanism underlying the enhanced cytotoxicity observed with combined berberine and etoposide treatment, γH2AX immunofluorescence staining was performed to evaluate DNA damage in A431 cells. Quantification of fluorescence intensity revealed that etoposide alone (10 μM) significantly increased γH2AX signal compared to the control group, reflecting elevated levels of DNA double-strand breaks. In contrast, berberine alone (10 μM) did not induce a significant increase in γH2AX intensity, suggesting a lack of direct genotoxicity. Notably, the combination of berberine and etoposide (10 μM each) resulted in a marked elevation of γH2AX levels, exceeding the effect of etoposide alone (Figure 6A,B). These findings suggest that berberine may potentiate etoposide-induced DNA damage not by directly inducing DNA breaks, but rather by interfering with cellular DNA repair mechanisms, consistent with previous reports showing that berberine induces oxidative DNA damage and suppresses homologous recombination repair.^31^

**Figure 6:**
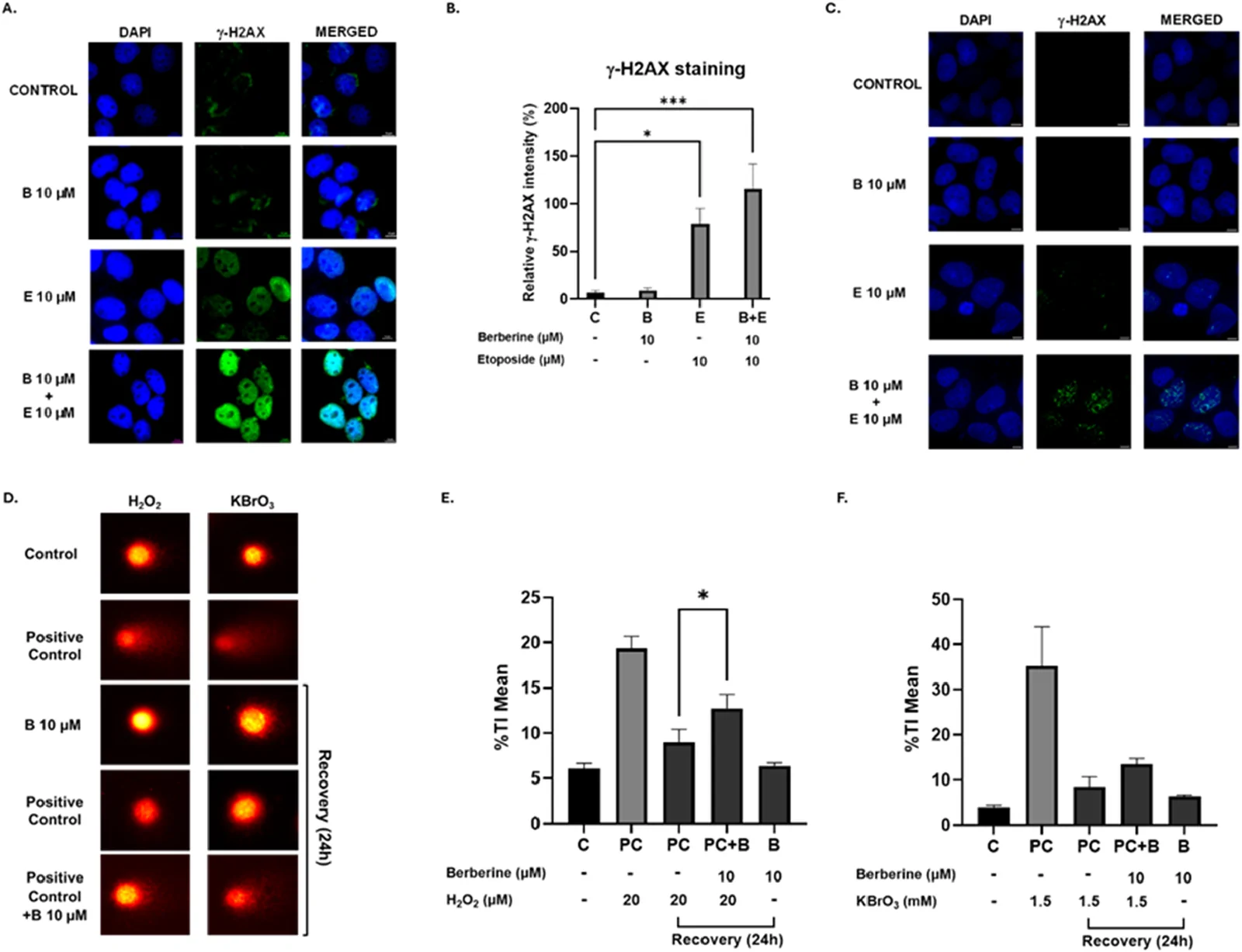
DNA damage induction and repair were evaluated in A431 cells following berberine treatment, alone or in combination with etoposide or oxidative DNA-damaging agents, using γH2AX immunofluorescence staining and Fpg-modified alkaline comet assays. (A) Representative γH2AX (green) immunofluorescence images of A431 cells treated with berberine (10 μM), etoposide (10 μM), or their combination for 24 h. Nuclei were counterstained with DAPI (blue). Scale bars: 10 μm. (B) Quantification of γH2AX fluorescence intensity corresponding to panel (A). Data are presented as mean ± SEM of three independent experiments, and statistical analysis was performed using one-way ANOVA and Dunnett’s post hoc test. *p ≤ 0.05, ***p ≤ 0.001. (C) Evaluation of DNA damage recovery following drug removal. After 24 h of treatment, cells were incubated in drug-free medium for an additional 24 h, and the γH2AX signal was analyzed by immunofluorescence. Scale bars: 10 μm. (D) Representative images of the Fpg-modified comet assay assessing recovery from oxidative DNA damage in A431 cells treated with berberine, hydrogen peroxide (H_2_O_2_), or potassium bromate (KBrO_3_). Quantification of comet tail intensity following (E) H_2_O_2_ or (F) KBrO_3_ treatment, alone or in combination with berberine. C: Control, B: Berberine, PC: Positive Control, E: Etoposide. Data are presented as mean ± SEM of three independent experiments, and statistical analysis was performed using one-way ANOVA and Sidak’s post hoc test. *p ≤ 0.05.

To determine whether berberine affects the resolution of etoposide-induced DNA damage, a recovery experiment was performed. A431 cells were treated with berberine (10 μM), etoposide (10 μM), or their combination for 24 h, followed by drug removal and an additional 24 h of incubation in drug-free medium. Immunofluorescence analysis revealed that γH2AX levels largely returned to baseline levels in etoposide-treated cells, indicating effective DNA damage repair. In contrast, cells treated with the berberine–etoposide combination retained elevated γH2AX signals after the recovery period, suggesting persistent DNA damage and delayed repair (Figure 6C).

To further characterize the contribution of berberine to DNA damage processing, oxidative DNA base lesions were assessed using the Fpg-modified comet assay. H_2_O_2_ and KBrO_3_ induced substantial oxidative DNA damage, which was efficiently repaired after a 24 h recovery period. In contrast, cotreatment with berberine delayed the repair, resulting in elevated DNA damage levels after recovery compared with the ox stress-generating agent alone. Notably, berberine alone did not cause sustained oxidative DNA damage, as post-recovery levels of DNA damage returned to those of the control group, confirming that berberine does not primarily act by inducing oxidative DNA lesions, but rather limiting the efficient processing and repair of ox DNA damage (Figure 6D–F). Together with the sustained γH2AX signaling observed during the recovery phase, these results support a role for berberine in impairing DNA damage repair, thereby exacerbating genotoxic stress in A431 cells.

### PCSK9 Inhibition and Impaired Resolution of DNA Damage Influence the Extent of the Synergistic Response to Berberine and Etoposide

To determine whether effective PCSK9 suppression is required for the synergistic interaction between berberine and etoposide, we employed palmatine, a structurally related protoberberine alkaloid. Unlike berberine, palmatine contains two methoxy groups (−OCH_3_) at positions 2 and 3, replacing the methylenedioxy moiety. Cell viability assays demonstrated that palmatine exhibited lower cytotoxicity than berberine in A431 cells, with even the highest tested concentration (100 μM) inducing less than 50% reduction in cell viability (Figure 7A). However, despite its structural similarity to berberine, this alkaloid showed markedly lower efficacy in reducing PCSK9 levels, as determined by ELISA analysis (Figure 7B), thereby reinforcing previous findings on the different effectiveness of berberine derivatives in suppressing PCSK9 expression.^32^ When palmatine was combined with etoposide under the same conditions as shown in Figure 7C, Bliss and HSA scores were −1.281 and 4.467, respectively, which implies a weaker synergistic effect compared to the berberine and etoposide combination. These findings indicate that a reduced inhibition of PCSK9 may explain the decrease in synergism when etoposide is combined with palmatine. To validate our observation that delayed repair of DNA damage is also critical for the manifestation of the synergistic effect, we employed doxorubicin, which has a mechanism of action similar to etoposide.^40,41^ A previous study reported that inhibition of the double-strand break repair protein RAD51 potentiates the cytotoxic efficacy of doxorubicin.^42^ Given that berberine has also been reported to reduce RAD51 levels,^31^ we hypothesized that this could enhance doxorubicin-induced cytotoxicity. MTT (Figure 7D,7E) and LDH assays (Figure 7F) confirmed the benefit of combining these compounds and further supported the inhibition of DNA damage repair as an important mechanism contributing to their synergistic effect.

**Figure 7:**
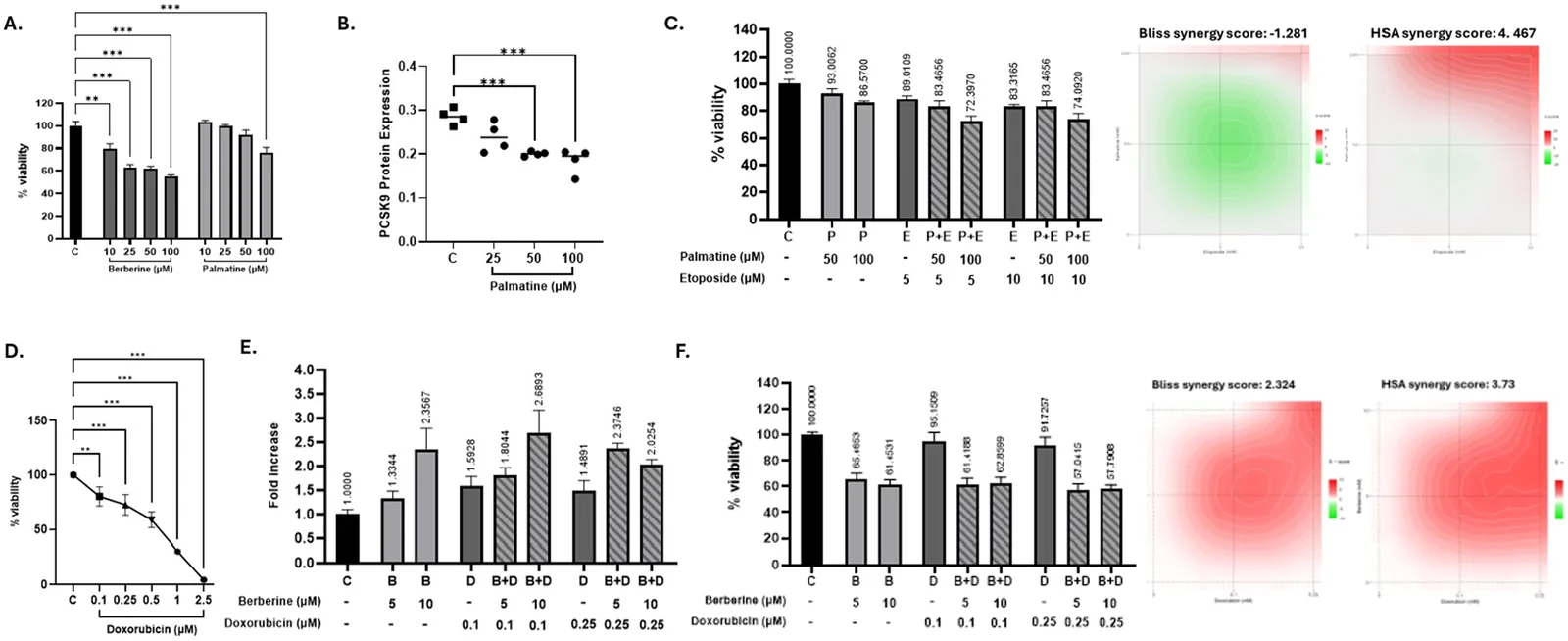
(A) Effects of the berberine and palmatine 10, 25, 50, 100 μM treatments on the cell viability of A431 cell line, (B) effects of 25, 50, 100 μM palmatine treatment on PCSK9 protein expression levels in A431 cell line, (C) effects of the combination treatment of etoposide with palmatine on the viability of A431 cells and the assessment of potential synergistic effects using the Bliss and HSA synergy scores; treatments of palmatine alone (50, 100 μM), etoposide alone (5, 10 μM), and the combination treatment of palmatine and etoposide are shown, (D) effects of the doxorubicin 0.1, 0.25, 0.5, 1, 2.5 μM treatments on the cell viability of A431 cell line, Effects of the combination treatment of doxorubicin with berberine on the viability of A431 cells and the assessment of potential synergistic using the Bliss and HSA synergy scores; Treatment of berberine alone (5, 10 μM), doxorubicin alone (0.1, 0.25 μM), and the combination treatment of berberine and doxorubicin determined by (E) LDH Assay, and (F) MTT Assay and the assessment of potential synergistic effects using the Bliss and HSA synergy scores. All treatments are performed for 24 h. C: Control, B: Berberine, P: Palmatine, E: Etoposide, D: Doxorubicin. Data are presented as mean ± SEM of three (A, B, C, D, E) or four (F) independent experiments, and statistical analysis was conducted using one-way ANOVA and Dunnett’s post hoc test. *p ≤ 0.05, **p ≤ 0.01, ***p ≤ 0.001.

### PCSK9 Silencing Enhances Etoposide-Induced Cytotoxicity in A431 Cells

To confirm the role of PCSK9 inhibition in enhancing etoposide-induced cytotoxicity, we employed genetic suppression of PCSK9. To this end, A431 cells were transfected with PCSK9-specific siRNA, and silencing efficiency was confirmed at the mRNA level (Figure 8A). Functional assessment revealed that PCSK9 silencing significantly enhanced the cytotoxicity of 10 μM etoposide compared with control siRNA–transfected cells (Figure 8B). Notably, the reduction in cell viability observed upon PCSK9 silencing paralleled the sensitizing effect previously detected with berberine treatment. These findings indicate that PCSK9 suppression is crucial to enhance cellular sensitivity to etoposide and support a functional role for PCSK9 in modulating the combinatorial response. These data raise the possibility that berberine, through modulation of PCSK9, may serve as a potential adjuvant to enhance the efficacy of DNA-damaging chemotherapeutics such as etoposide.

**Figure 8:**
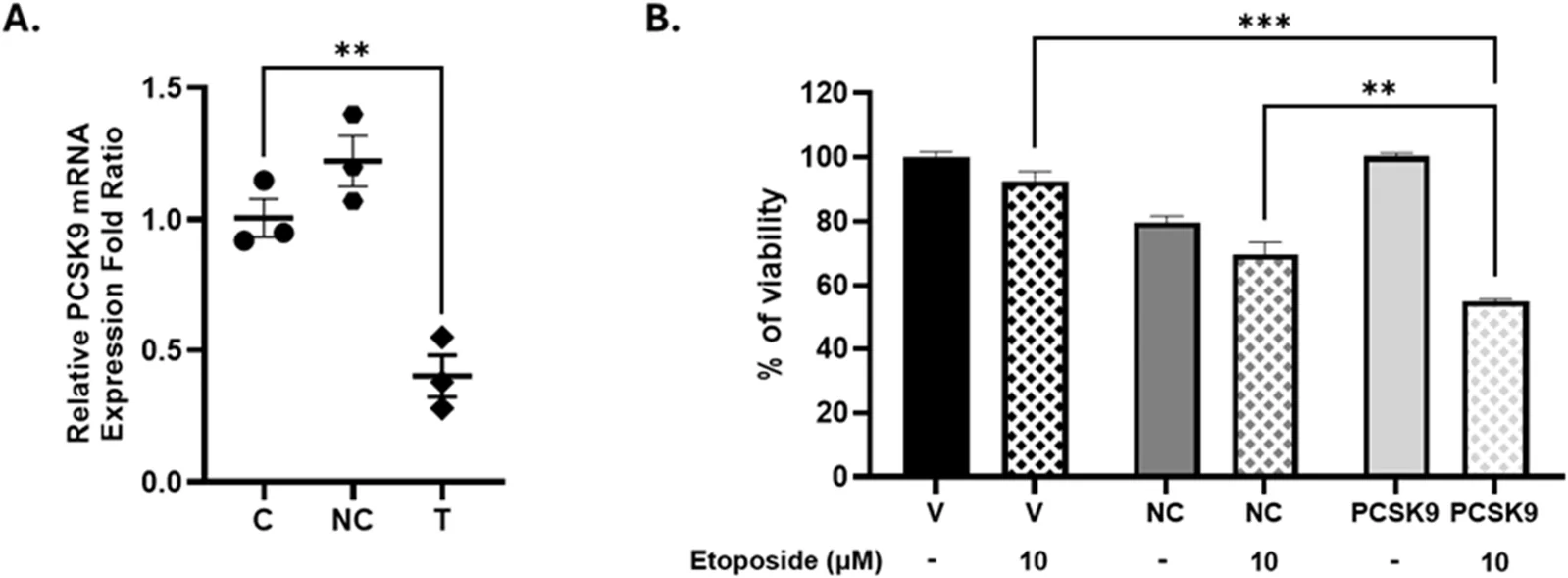
PCSK9 silencing mimics the effect of berberine on etoposide-induced cytotoxicity. (A) Validation of PCSK9 silencing efficiency in A431 cells transfected with control siRNA (NC) or PCSK9-specific siRNA (T). Data are presented as mean ± SEM of three independent experiments. Statistical analysis was performed using one-way ANOVA followed by Dunnett’s post hoc test. **p ≤ 0.01. (B) Effects of PCSK9 silencing on the viability of A431 cells following etoposide treatment. Cells were transfected with control siRNA or PCSK9-specific siRNA and subsequently treated with 10 μM etoposide. Cell viability was assessed at 24 h by using the MTT assay. C: Control (only medium), V: Vehicle (only lipofectamine), NC: Negative Control (control siRNA), T: Transfection Group (PCSK9-specific siRNA). Data are presented as mean ± SEM of three independent experiments. Statistical analysis was performed using one-way ANOVA followed by Sidak’s post hoc test. **p ≤ 0.01, ***p ≤ 0.001.

### Berberine and Etoposide Exert Synergistic Effects in SK-MEL-28 Skin Cancer Cells

To further broaden the significance of our findings, we utilized SK-MEL-28, a human malignant melanoma cell line, widely used in research to test traditional cytotoxic chemotherapies.^43,44^ In this cell line, both etoposide and berberine reduced cell viability in a dose-dependent manner (Figure 9A) while basal PCSK9 protein expression was higher in A431 cells compared to SK-MEL-28 cells (Figure 9B). However, ELISA analysis confirmed that also in this cell line, berberine was able to reduce PCSK9 levels in a dose-dependent manner (Figure 9C). Combinatory treatment of berberine and etoposide in SK-MEL-28 cells resulted in positive synergy values (Bliss score 2.971 and HSA score 13.28) similar to the ones observed in A431 cells (Figure 9D). Collectively, these results support our hypothesis that effective PCSK9 suppression may be a key determinant of combinatory sensitization in skin cancer cells. Further studies will be required to unveil the exact mechanisms underlying the relationship between PCSK9 inhibition and the enhanced sensitivity of cancer cells to specific chemotherapeutic agents.

**Figure 9:**
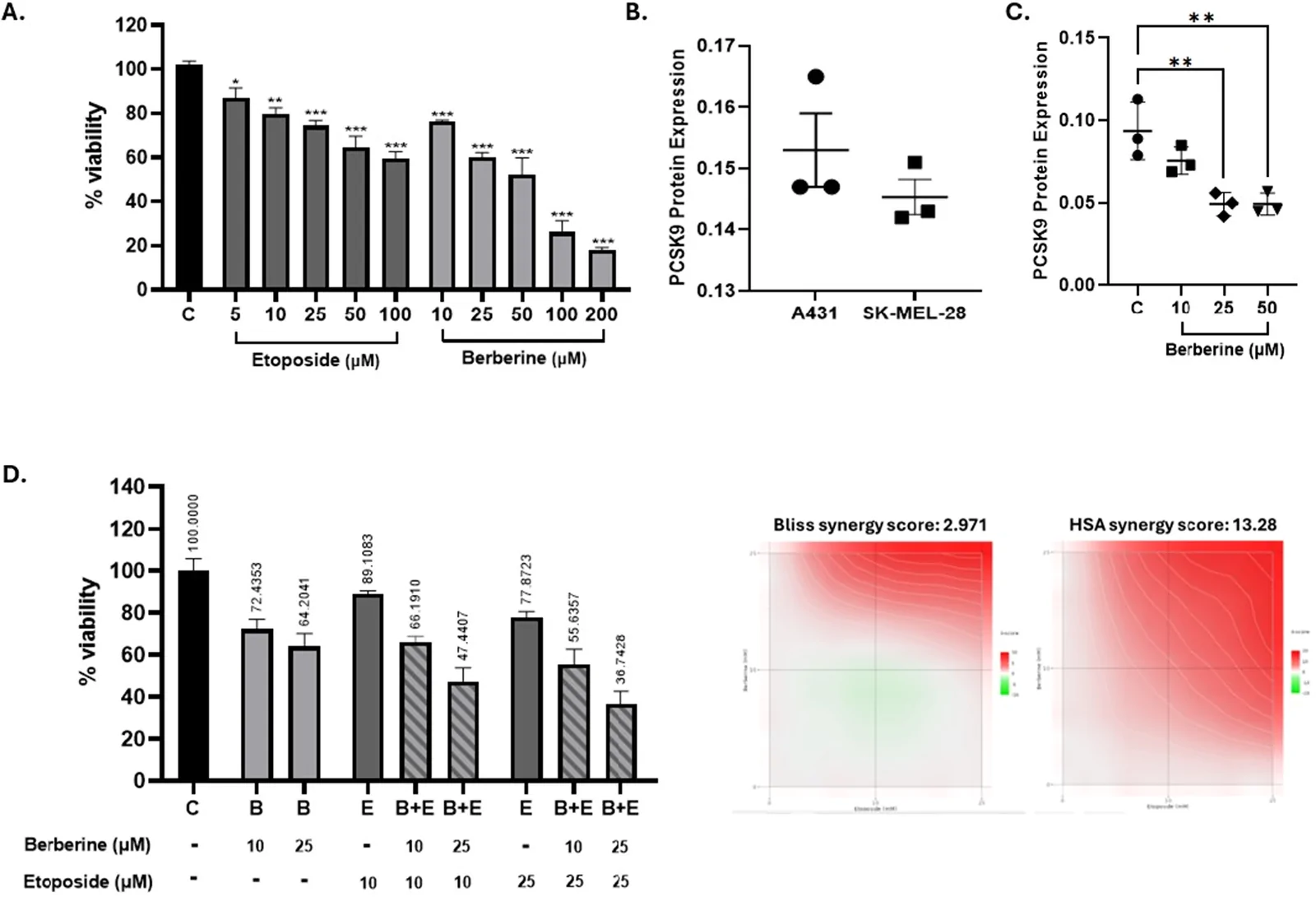
(A) Effects of the etoposide (5, 10, 25, 50, 100 μM) and berberine (10, 25, 50, 100, 200 μM) treatments on the cell viability of SK-MEL-28 cell line, (B) PCSK9 protein expression levels of A431 and SK-MEL-28 cell lines, (C) effects of the berberine (10, 25, 50 μM) treatments on PCSK9 protein expression levels of SK-MEL-28 cell line, (D) effects of the combination treatment of etoposide with berberine on the viability of SK-MEL-28 cells and the assessment of potential synergistic effects using the Bliss and HSA synergy scores; Treatment of berberine alone (10, 25 μM), etoposide alone (10, 25 μM), and the combination treatment of berberine and etoposide. All treatments are performed for 24 h. C: Control, B: Berberine, E: Etoposide. Data are presented as mean ± SEM of three independent experiments, and statistical analysis was performed using one-way ANOVA and Dunnett’s post hoc test. *p ≤ 0.05, **p ≤ 0.01, ***p ≤ 0.001.

## Discussion

Cancer and CVD have been viewed as separate pathological conditions, with their primary link being the cardiotoxic effects of certain cancer therapies, which elevate the risk of cardiac complications. However, several clinical studies indicate a bidirectional relationship: individuals with cancer have a higher risk of developing CVD, while those with CVD are more susceptible to cancer.^45,46^ Indeed, several pathological conditions are shared between these two diseases, including altered lipid metabolism, increased oxidative stress, and metabolic dysregulation.^47,48^ PCSK9 is an important protein involved in lipid metabolism, primarily influencing blood cholesterol levels by regulating the number of LDL receptors on liver cell surfaces. However, recent studies have revealed that PCSK9 is also involved in the regulation of key cellular processes in cancer cells.^1,49^ High PCSK9 levels correlate with a poor prognosis in various types of cancer: hepatocellular carcinoma, breast, lung, and gastric cancer.^50,51^ Thus, PCSK9 can promote cancer cell proliferation by increasing cyclin expression and activation of mitogen-activated protein kinase (MAPK) or phosphatidylinositol 3-kinase/protein kinase B (PI3K/Akt).^52,53^ Additionally, PCSK9 has been linked to cancer via dysregulation of apoptosis and immune response.^33,54,55^ In this work, we examined PCSK9 levels in a panel of cancer cell lines and observed that the PCSK9-overexpressing A431 cell line is particularly sensitive to berberine-induced cytotoxicity. Berberine, an isoquinoline alkaloid found in the roots, rhizomes, and stem bark of several plants, has been shown to be a potent inhibitor of PCSK9 levels.^15,36^ Our quantitative RT-PCR analysis revealed a dose-dependent decrease in PCSK9 expression, which correlated with increased LDLR levels as assessed by immunofluorescence staining. The use of berberine in combination with etoposide increases cytotoxicity in A431 cancer cells and reduces their migration potential, as measured by a wound-healing assay. Similar beneficial effects have been observed with berberine in combination with other chemotherapeutic drugs,^19,20,56,57^ but to our knowledge, the combination treatment with etoposide has not yet been investigated. Synergism between berberine and etoposide was identified using SynergyFinder software, which indicated the strongest interaction at a concentration of 10 μM for both compounds, especially when the HSA score was employed. Immunofluorescence analysis of the DNA damage response using the γ-H2AX antibody showed that berberine alone did not significantly affect γ-H2AX staining. However, the combination of berberine and etoposide induced a significant increase in γ-H2AX intensity compared to etoposide alone. This led us to hypothesize that berberine does not directly cause DNA damage at the tested concentration but may be involved in the disruption of intracellular DNA repair mechanisms, as suggested by previous studies.^31,58^ To test this hypothesis, we conducted recovery experiments in which cells were treated with berberine, etoposide, or their combination for 24 h, followed by a 24 h recovery period after medium replacement. γ-H2AX analysis showed that DNA damage persisted only in A431 cells treated with the combination of berberine and etoposide, whereas cells treated with etoposide alone exhibited complete recovery. When oxidative DNA base lesions were assessed using the Fpg-modified comet assay, cotreatment with berberine enhanced the persistence of DNA damage induced by hydrogen peroxide and potassium bromate, confirming that berberine plays a role in interfering with DNA repair processes. While the berberine-induced impairment of DNA repair is supported by several previous studies,^31,58,59^ there is currently no direct evidence demonstrating that PCSK9 inhibition contributes to the synergistic effects of berberine with chemotherapeutic drugs. Therefore, to substantiate our hypothesis that the berberine-induced increase in sensitivity to etoposide is mediated, at least in part, by PCSK9 inhibition, we employed two additional approaches using palmatine and a specific PCSK9 siRNA. Palmatine, an isoquinoline alkaloid structurally very similar to berberine, is a less effective inhibitor of PCSK9,^32^ while specific PCSK9 siRNA approaches were already used to investigate the effects of PCSK9 on cell proliferation and induction of apoptosis.^54,60^ In our experiments, while palmatine association with etoposide was associated with a decrease in synergy scores, the PCSK9 inhibition by siRNA led to a significant increase in etoposide-induced cytotoxicity, confirming the relevance of PCSK9 in modulating cancer cell sensitivity to chemotherapy. However, while PCSK9 expression is increased and linked to poorer outcomes in certain malignancies, in other cancer types it shows an inverse association, underscoring the need for further preclinical and clinical studies to clarify its role in tumor progression. Furthermore, in evaluating the role of PCSK9 in cancer, its effects on lipid metabolism must also be taken into account, as PCSK9 is a key regulator of LDL receptor degradation and systemic cholesterol homeostasis. By modulating circulating LDL cholesterol levels and cellular cholesterol uptake, PCSK9 can influence membrane synthesis, lipid raft formation, and oncogenic signaling pathways that are critical for tumor cell proliferation and survival.

## Experimental Section

### Chemicals and Reagents

The chemotherapeutic drugs etoposide, cisplatin, sorafenib and doxorubicin, as well as, berberine chloride, palmatine chloride, DMSO, Acridine Orange, Ethidium Bromide, Potassium Bromate (KBrO_3_), and 3-[4,5-dimethylthiazol-2-yl]-2,5-diphenyltetrazolium bromide (MTT) were all purchased from Sigma-Aldrich (Munich, Germany), while trypsin/EDTA and Lipofectamine 3000 were from Thermo Fisher Scientific (Waltham, MA, USA). Berberine chloride (catalog number B3251) has a reported purity (TLC) of 99%, while palmatine chloride (catalog number SMB00472) has a reported purity (HPLC) of 99%. Low-melting-point agarose (LMP), normal-melting-point agarose (NMP), Na_2_EDTA, Tris HCl, and Triton X-100 were all from Carl Roth GmbH & Co (KG, Karlsruhe, Germany). Bovine serum albumin (BSA) was purchased from GeneON Biosciences (Germany). Formamidopyrimidine DNA glycosylase (Fpg) was obtained from New England Biolabs (Ipswich, Massachusetts, USA). PCSK9-targeting siRNA (sc-45482) and conjugated control siRNA-A (sc-37007) were purchased from Santa Cruz Biotechnology (Dallas, TX, USA).

### Cell Culture

The following immortalized cell lines were selected in this study: HepG2 (hepatocellular carcinoma, RRID: CVCL_0027), A431 (human epidermoid carcinoma, RRID: CVCL_0037), HCT116 (colorectal carcinoma, RRID: CVCL_0291), PC-3 (prostate carcinoma, RRID: CVCL_0035), and SK-MEL-28 (cutaneous melanoma, RRID: CVCL_0526).

All cell lines were from American Type Culture Collection (Manassas, VA, USA) and were maintained in DMEM + GlutaMAX medium supplemented with 10% fetal bovine serum (FBS), 100 IU/mL penicillin, and 100 μg/mL streptomycin (all from Thermo Fisher Scientific) in a humidified incubator at 37 °C and 5% CO_2_. Cells were routinely tested for mycoplasma contamination using a Mycoplasma detection kit (InvivoGen, Toulouse, France). For all the experiments, unless different conditions are specified, cell seeding was performed by using 10% FBS DMEM, which was replaced with 1% FBS DMEM before the treatments, including the control groups.

### MTT Assay

The MTT assay was performed to evaluate the cytotoxicity of berberine and selected chemotherapeutic drugs, as previously described.^61^ 3-(4,5-dimethylthiazol-2-yl)–2,5-diphenyltetrazolium bromide (MTT) is reduced to formazan by NAD­(P)­H-dependent oxidoreductase enzymes in viable cells. Briefly, tumor cell lines were treated with the selected concentrations of berberine alone or in combination with chemotherapeutic drugs in DMEM containing 1% FBS to reduce baseline metabolic activity. Control groups were also maintained in DMEM containing 1% FBS during the experimental time period. One hour before the end of the incubation period, the supernatants were collected (for LDH assay), and MTT diluted in DMEM was added. MTT was subsequently removed, and the cells were lysed with DMSO. The optical density (OD) was then measured at 570 nm using an Infinite M200 PRO spectrometer (Tecan, Männedorf, Switzerland).

### LDH Assay

LDH release in the culture medium was determined using a CyQuant LDH Cytotoxicity assay (Thermo Fisher Scientific). Increased LDH release into the supernatant generally corresponds to a decrease in viable cell number, reflecting loss of membrane integrity, and can be detected by measuring absorbance at 490 and 680 nm. To determine LDH activity, the absorbance measured at 680 nm (background) was subtracted from that recorded at 490 nm. The results are reported as fold changes in LDH levels relative to control cells.

### Acridine Orange/Ethidium Bromide Staining

For cytotoxicity evaluation, dual acridine orange-ethidium bromide staining (AO/EB) was also employed, according to a modified protocol from Liu et al.^62^ Briefly, AO/EB dye mix was prepared by combining 1 μL each of ethidium bromide (EB) (3 mg/mL) and acridine orange (AO) (5 mg/mL) with 1 mL of PBS. After, 2 μL of the resulting AO/EB mix was added to 20 μL of cell suspension, placed on clean microscope slides, covered with coverslips, and examined using a 20× objective on a fluorescence microscope. AO permeates all cells, intercalates into double-stranded DNA, and, upon 488 nm excitation, emits a green fluorescence. EB is internalized only by dead cells, when cytoplasmic membrane integrity is impaired, and stains the nucleus red.^63^ As EB fluorescence predominates over AO fluorescence, viable cells exhibit green nuclei, whereas late apoptotic or dead cells display bright orange to red nuclei. At least 100 cells for each treatment condition were scored to determine the percentage of orange/red (dead) versus green (viable) cells.

### ELISA

ELISA assays were performed using a human PCSK9 ELISA kit (Abnova, Aachen, Germany) according to the manufacturer’s protocol. To evaluate intracellular levels of PCSK9 in cells lysed in RIPA buffer, protein samples were equilibrated using the BCA Protein Assay Kit (Thermo Fisher Scientific).

### Immunofluorescence Staining

Cells were seeded on sterile coverslips and fixed with 4% paraformaldehyde for 15 min at room temperature. Nonspecific binding was blocked using 2% BSA in PBS for 1 h at room temperature. After blocking, cells were incubated overnight at 4 °C with a monoclonal rabbit anti-LDLR primary antibody (RRID: AB_3069742, Thermo Fisher Scientific). After the incubation, cells were washed 3 times with PBS and incubated with a goat antirabbit Alexa Fluor-conjugated secondary antibody (RRID: AB_143165, Thermo Fisher Scientific) for 1 h at room temperature in the dark. Cell membranes were then permeabilized using 0.1% Triton X-100 for 10 min. DAPI (Thermo Fisher Scientific) (1 μg/mL) was used to stain nuclei. Images were captured using a Zeiss Axio Observer Z1. LDLR expression was quantified using ImageJ software. For DNA damage detection, cells were permeabilized with Triton X-100 and blocked with 2% BSA before incubation with the primary antibody for γ-H2AX (Ser139) (RRID: AB_2810155, Thermo Fisher Scientific).

### Gene Expression Analysis

RNA was extracted from A431 cell lines using TRIzol lysis following a reported protocol.^64^ The RNA was subsequently converted into cDNA using a High-Capacity cDNA Reverse Transcription Kit (Thermo Fisher Scientific). Real-time PCR was performed using the QuantStudio Real-Time PCR System (Thermo Fisher Scientific) with the SYBR green reaction mixture (Thermo Fisher Scientific). The RNA concentration was measured using a NanoDrop device (Thermo Fisher Scientific). Primers designed using the Primer Blast tool were supplied by Nano-Diagnostika (Vilnius, Lithuania). The sequences of the primers were:

(1) GAPDH (FW-GTCTCCTCTGACTTCAACAGCG; RV-ACCACCCTGTTGCTGTAGCCAA);
(2) PCSK9 (FW-GACACCAGCATACAGAGTGACC; RV-GACACCAGCATACAGAGTGACC);
(3) LDLR (FW–GAATCTACTGGTCTGACCTGTCC; RV–GGTCCAGTAGATGTTGCTGTGG).

### Wound Healing Assay

A431 cells were seeded in 12-well plates and cultured overnight. After achieving full confluency, the cell monolayer was mechanically scratched using a sterile pipette tip, washed with PBS, and then treated with berberine and etoposide alone and in combination. Images were acquired at 0, 3, 6, 12, and 24 h using a Leica Eclipse Ti inverted microscope (Wetzlar, Germany) with 4× magnification. Change in scratch area upon wound closure at each time point was calculated using ImageJ software (RRID: SCR_003070).

### Fpg-Modified Comet Assay

The alkaline comet assay allows the detection of single- and double-stranded DNA breaks. Incorporation of an additional step treating DNA with formamidopyrimidine DNA glycosylase (Fpg)–a DNA glycosylase that specifically recognizes and removes oxidized purines (e.g., 8-oxoguanine and formamidopyrimidines)–enables detection of an additional oxidative DNA damage.^65^ The Fpg-modified comet assay was performed according to Azquetta et al.,^66^ with minor adjustments, and is reported according to MIRCA guidelines.^67^ Briefly, 40 μL of cell suspension (2 × 10^5^ cells/mL) was gently mixed with 40 μL of 1% low-melting-point (LMP) agarose at 37 °C (final LMP concentration, 0.5%). Two 80 μL drops of this mixture were pipetted into a microscope slide, precoated with 1% normal melting point (NMP) agarose and covered with a coverslip (24 mm × 24 mm) to create a 2-gel slide. Slides were incubated at 4 °C for 10 min to facilitate gel solidification. The coverslips were carefully removed, after which the slides were immersed in cold lysis solution (2.5 M NaCl, 0.1 M Na_2_EDTA, 10 mM Tris-HCl, pH 10, with 1% Triton X-100 and 10% DMSO added immediately prior to use) for 90 min at 4 °C. After that, slides were washed three times with cold enzyme reaction buffer (40 mM HEPES, 0.1 M KCl, 0.5 mM Na_2_EDTA, 0.2 mg/mL BSA, pH 8.0) for 5 min each at 4 °C, and then placed on a cold metal plate on ice. Next, 50 μL of Fpg enzyme solution (20 U/mL) was applied to each gel, covered with coverslips, and incubated in a humid chamber for 30 min at 37 °C. The enzymatic reaction was terminated by placing the slides on a cold metal plate on ice and removing the coverslips. Slides were then placed side by side in the horizontal gel electrophoresis tank COMET-20 SYSTEM (Scie-Plas, Cambridge, UK), filled with cold, freshly prepared electrophoresis buffer (1 mM Na_2_EDTA, 0.3 M NaOH, pH 13) and left for 20 min at 4 °C for the DNA to unwind. Electrophoresis was performed at 0.9 V/cm for 21 min at 4 °C. To maintain the stable temperature of the buffer, the buffer was additionally cooled and circulated. Later slides were washed with a cold PBS for 10 min at 4 °C. Finally, slides were stained with 1 μg/mL DAPI (80 μL per gel) and stored in a dark place at 4 °C at least overnight, until analysis. Slides were examined by a single scorer using a fluorescence microscope (Nikon Eclipse 80i, Nikon, Tokyo, Japan) at 400× magnification. Image capture and analysis were performed using LUCIA Comet AssayTM software version 7.60 (Laboratory Imaging, s.r.o., Czech Republic). 50 randomly chosen nucleoids per gel were scored (a total of 100 comets per sample). Tail Intensity (% TI) was used as an indicator of DNA damage. The overall level of DNA migration was expressed as the mean value of %TI ± SEM.

### PCSK9 Gene Silencing by siRNA

A431 cells were maintained in DMEM supplemented with 10% FBS and seeded at a density of 1.5 × 10^5^ cells per well in 6-well plates 1 day prior to transfection, reaching approximately 40–50% confluency at the time of transfection. siRNA-mediated gene silencing was performed using Lipofectamine 3000 with PCSK9-targeting siRNA or conjugated control siRNA. Prior to transfection, cells were washed once with PBS and incubated in serum- and antibiotic-free Opti-MEM medium. siRNA–lipid complexes were prepared by diluting the indicated amount of siRNA (100 pmol per well) in Opti-MEM and combining it with Lipofectamine 3000 diluted separately in Opti-MEM. The mixtures were incubated at room temperature for 20 min to facilitate complex formation and were subsequently added dropwise to the cells. Cells were incubated with the transfection complexes under serum-free conditions for 4 h, after which an equal volume of antibiotic-free growth medium containing 20% FBS was added directly to each well without removing the transfection mixture, resulting in a final serum concentration of 10%. Cells were further incubated under standard culture conditions and harvested 24 h after transfection for downstream analyses, including RNA isolation. Untreated cells and scrambled siRNA-transfected cells were included as controls in all experiments.

### Drug Interaction Assessment

SynergyFinder (version 3, RRID:SCR_019318) is an online tool used to assess drug–drug interactions. In this study, it was used to evaluate the interactions of berberine in combination with etoposide, sorafenib, or cisplatin. Dose–response data were obtained from MTT assays performed in A431 cells. SynergyFinder employs multiple models for the assessment of drug synergy, and in our case, the Bliss Independence and Highest Single Agent (HSA) models were used. The Bliss Independence model assesses the scenario where the two drugs act independently. It predicts the expected effect based on the probability that unrelated events occur simultaneously. If the observed effects of the combination exceed this expected value, it indicates a synergistic interaction. On the other hand, the HSA model evaluates the effects of the drug combination versus the maximum effects of the individual drugs. If the combination produces a greater effect than the most effective single agent, it suggests a synergistic interaction. In both models, synergism is indicated by a positive value.

### Statistical Analysis

Data are presented as arithmetic means ± s.e.m. of at least 3 independent experiments. Statistical analysis was conducted, and the data were graphically represented using GraphPad Prism version 10.2.0 (RRID: SCR_002798). Data were analyzed using a one-way ANOVA. Dunnett or Sidak multiple comparison tests were used to compare, respectively, each treatment vs control or preselected pairs of columns. Significance was ascribed at *p* ≤ 0.05 with *p ≤ 0.05, **p ≤ 0.01 and ***p ≤ 0.001.

## Supplementary Material



## Data Availability

All data associated with this study are presented in the paper or Supporting Information. All the supporting data for this study are available from the corresponding author on reasonable request.

## Notes

Experimental methods: effects of berberine on the gene expression levels of LDLR of A431 cells; effects of berberine on the nuclei of A431 cells; effects of chemotherapeutics (etoposide, sorafenib, cisplatin) on cell viability of A431 cells determined by MTT assay; effects of berberine, etoposide combination treatment on cell viability of A431 cells determined by acridine orange/ethidium bromide dual staining (PDF)

## Abbreviations

ACC: Acetyl-CoA Carboxylase
AMPK: Adenosine Monophosphate-activated Protein Kinase
AO: Acridine Orange
BCA: Bicinchoninic Acid
BSA: Bovine Serum Albumin
cDNA: complementary DNA
CVD: Cardiovascular Disease
DAPI: 4′,6-diamidino-2-phenylindole
DMEM: Dulbecco’s Modified Eagle Medium
DMSO: Dimethyl Sulfoxide
EB: Ethidium Bromide
FBS: Fetal Bovine Serum
γ-H2AX: phosphorylated H2AX
GAPDH: Glyceraldehyde 3-Phosphate Dehydrogenase
HSA: Highest Single Agent
LDH: Lactate Dehydrogenase
LDLR: Low-Density Lipoprotein Receptor
MAPK: Mitogen-Activated Protein Kinase
mTOR: Mammalian Target of Rapamycin
MHC-I: Major Histocompatibility Complex Class I Molecules
MTT: 3-(4,5-dimethylthiazol-2-yl)-2,5-diphenyltetrazolium bromide
NF-κB: Nuclear Factor kappa-light-chain-enhancer of activated B cells
PBS: Phosphate-Buffered Saline
PCSK9: Proprotein Convertase Subtilisin/Kexin type 9
PI3K/Akt: Phosphatidylinositol 3-kinase/Protein kinase B
RIPA buffer: Radioimmunoprecipitation assay buffer
siRNA: small interfering RNA
SCAP: Cholesterol Sensor Protein
SREBP2: Sterol Regulatory Element-Binding Protein 2
VEGF: Vascular Endothelial Growth Factor
