# Supplementary material for: Berberine and Etoposide Exert Synergistic Anticancer Effects in A431 Epidermoid Carcinoma Cells
Source: ACS Omega. 2026 Oct 2;11(40):61344–57. doi: 10.1021/acsomega.6c06421 (PMC13670754; doi:10.1021/acsomega.6c06421)
Supplement: Supplementary file 1 [file ao6c06421_si_001.pdf]

Berberine and Etoposide Exert Synergistic Anticancer Effects in A431 Epidermoid Carcinoma Cells

Zeynep Ozlem Cinar[1], Valerija Šatrova[1], Akshay Kumar Vijaya[2], Milda Babonaitė-Aleksiejūnė[3], Goda Glebavičiūtė[1], Aurelijus Burokas[2] and Giulio Preta[1,*]

[1]Institute of Biochemistry, Life Sciences Center, Vilnius University, LT-10257 Vilnius, Lithuania.

[2]Department of Biological Models, Institute of Biochemistry, Life Sciences Center, Vilnius University, LT-10257 Vilnius, Lithuania.

[3]Institute of Biosciences, Life Sciences Center, Vilnius University, LT-10257 Vilnius, Lithuania.

*Corresponding author: Dr. Giulio Preta giulio.preta@bchi.vu.lt

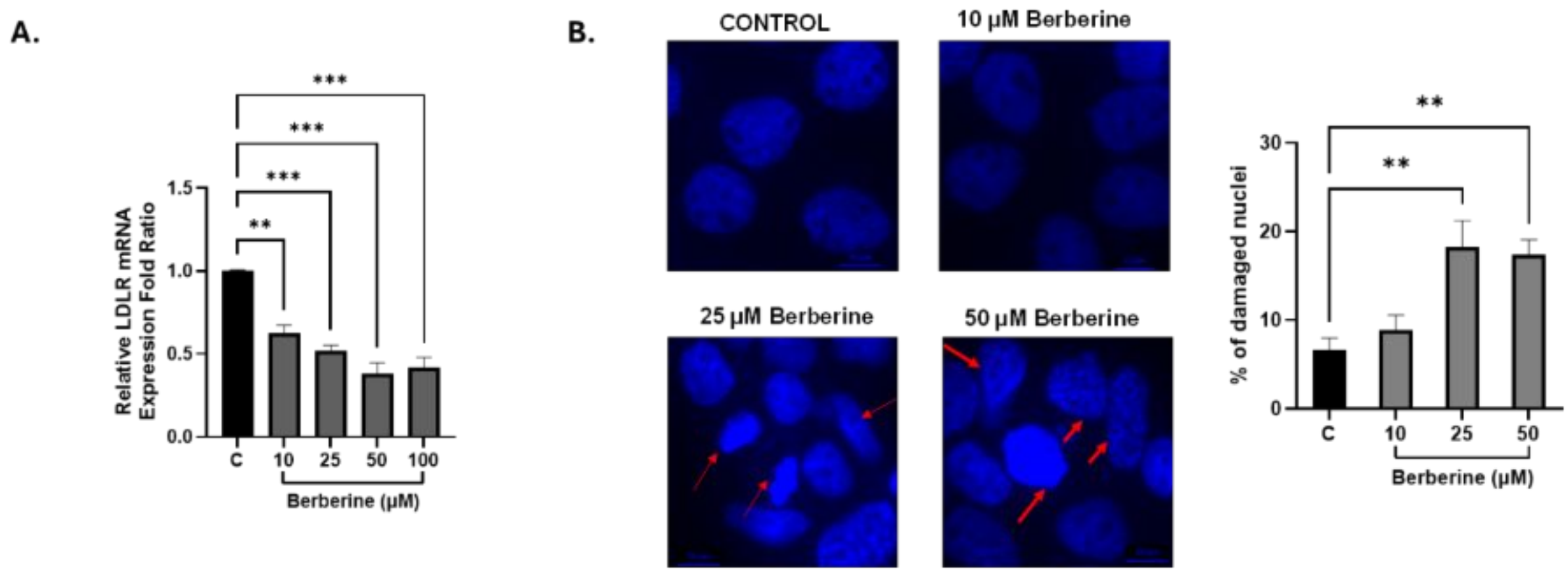

Figure S1. (A) Effects of berberine on LDLR at the mRNA level in A431 cells following berberine treatment at 10, 25, 50, 100 µM for 24 h. **(B)** Effects of berberine treatment (10, 25, 50 µM) on the nuclei of A431 cells at 24 hours were determined by DAPI staining. Representative immunofluorescence images showing nuclei, stained with DAPI (blue), and quantification of relative nuclear damage (%). Scale bars: 10 µm. C: Control. Data shown are mean ± SEM of four independent experiments, and statistical analysis was performed using one-way ANOVA and Dunnett's post hoc test. $^{**}p \leq 0.01$, $^{***}p \leq 0.001$.

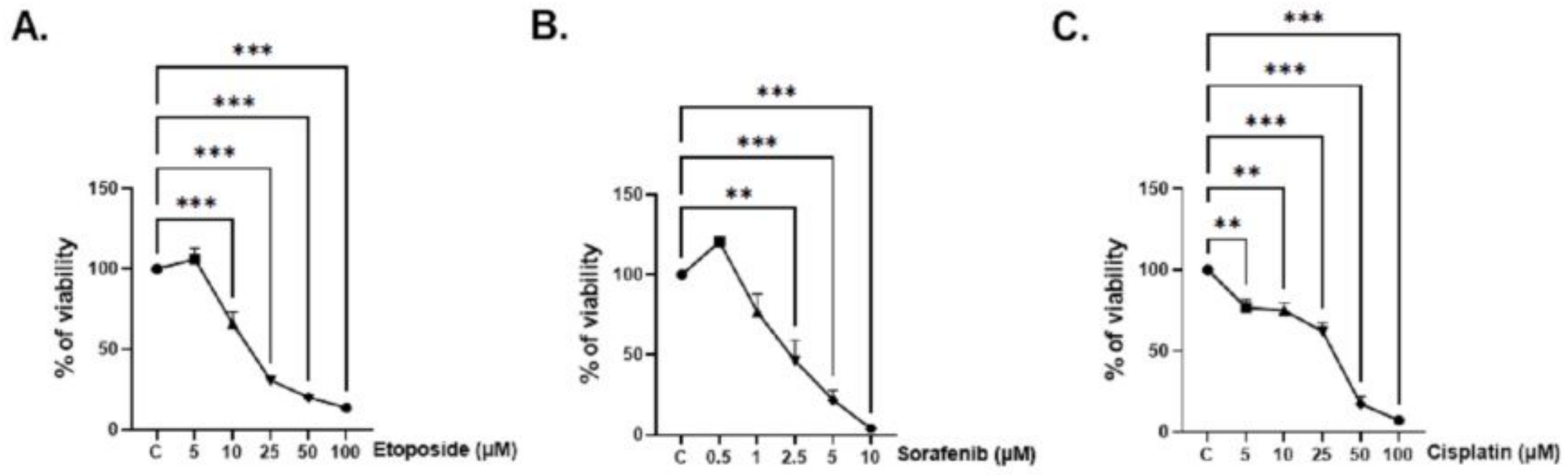

Figure S2. Effects of chemotherapeutics on the A431 cell line at 24 hours determined by MTT Cytotoxicity Assay. (**A)** Viability of A431 cells following 5, 10, 25, 50, 100 μM etoposide treatment. (**B)** Viability of A431 cells following 0.5, 1, 2.5, 5, 10 μM sorafenib treatment. (**C)** Viability of A431 cells following 5, 10, 25, 50, 100 μM cisplatin treatment. C: Control. Data shown are mean ± SEM of three (etoposide, cisplatin) or four (sorafenib) independent experiments, and statistical analysis was performed using one-way ANOVA and Dunnett's post hoc test. $^{**}p \leq 0.01$, $^{***}p \leq 0.001$.

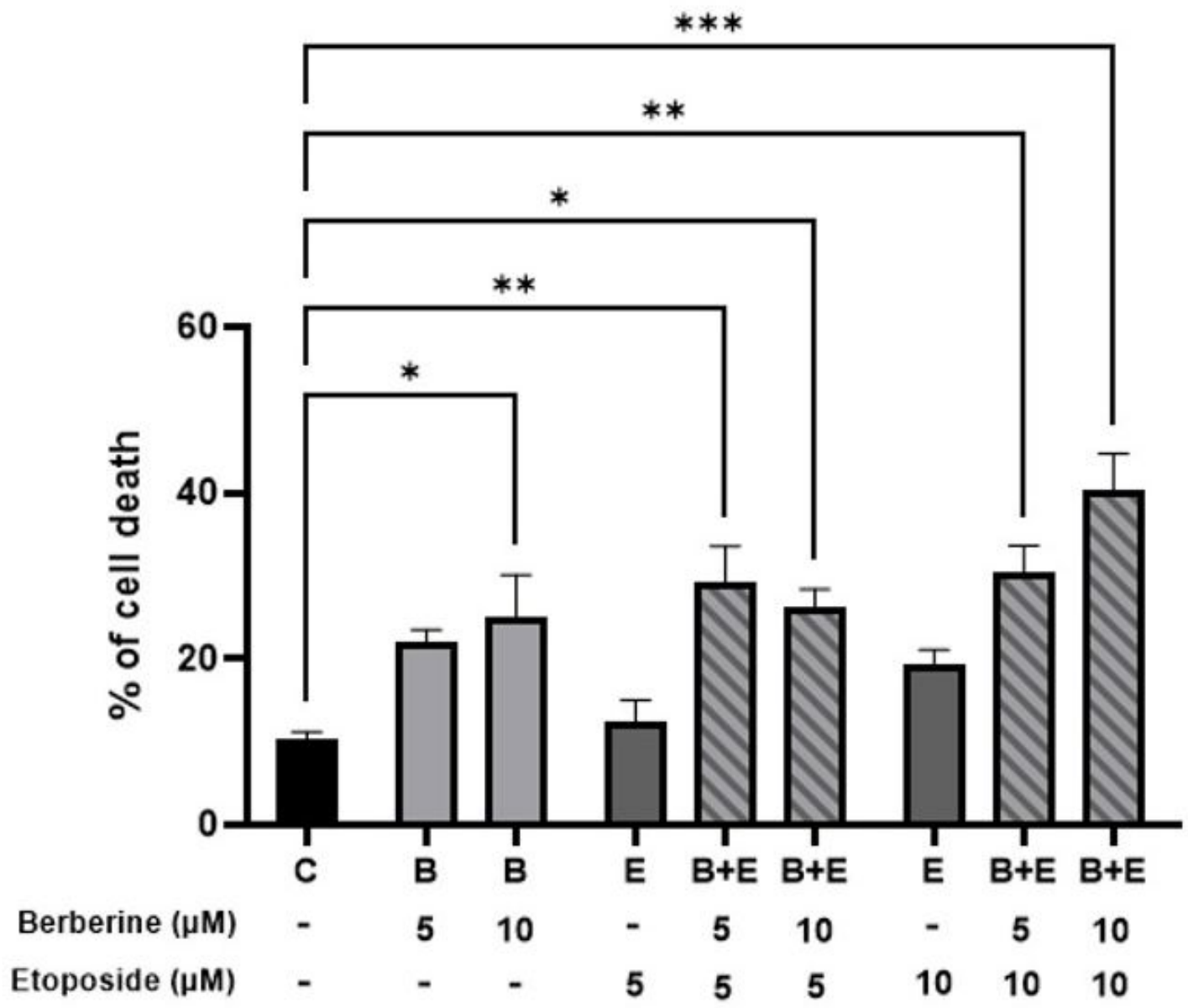

Figure S3. Effects of the combination treatment of etoposide with berberine on the viability of A431 cells at 24 hours, determined by acridine orange/ethidium bromide dual staining. Treatment of berberine alone (5, 10 µM), etoposide alone (5, 10 µM), and the combination treatment of berberine and etoposide. All possible combinations of the indicated concentrations were evaluated. C: Control, B: Berberine, E: Etoposide. Data shown are mean ± SEM of four independent experiments, and statistical analysis was performed using one-way ANOVA and Dunnett's post hoc test. *$p \leq 0.05$, **$p \leq 0.01$, ***$p \leq 0.001$.
